# Management of Large Proximal Ureteral Stones: A Comparative Clinical Trial Between Transureteral Lithotripsy (TUL) and Shock Wave Lithotripsy (SWL)

**DOI:** 10.5812/numonthly.3936

**Published:** 2012-06-20

**Authors:** Seyed Mohammadreza Rabani, Ali Moosavizadeh

**Affiliations:** 1 Beheshti Teaching Hospital, Yasouj University of Medical Sciences, Yasouj, IR Iran; 2 Epidmiology Department, Yasouj University of Medical Sciences, Yasouj, IR Iran

**Keywords:** Lithotripsy, High-Energy Shock Waves, Ureteral Calculi

## Abstract

**Background:**

A review of the related medical journals indicates that there is no definite evidence-based option for managing large proximal ureteral stones, although many procedures such as transureteral lithotripsy (TUL), shock wave lithotripsy (SWL), percutaneous nephrolithotripsy, laparoscopic ureterolithotomy, and open ureterolithotomy are currently used to treat this urological problem.

**Objectives:**

In this study, we tried to determine the most appropriate treatment plan for proximal ureteral stones larger than 12 mm by comparing the two most commonly used procedures.

**Patients and Methods:**

Between February 2005 and April 2011, 62 patients including 40 males and 22 females (mean age 39.5 years, range 19 to 64) with proximal ureteral stones larger than 12 mm (12–26 mm) with a mean size of 17.64 mm were prospectively divided into two groups consisting of 32 patients who underwent TUL (group A) and 30 who underwent SWL (group B). In unsuccessful cases, repeat SWL or TUL was planned. Patients who could not tolerate the lithotomy position, younger than 18 years, had undergone coagulopathy, had concurrent renal and ureteral stones, were pregnant, or had sepsis were excluded from this study.

**Results:**

Stone access was successful in 28 patients and the treatment was efficient in 18 patients (56.25%) in group A. For the patients with successful stone access but unsuccessful TUL, a DJ was inserted and a second ureteroscopic intervention was performed. The second intervention was successful in 7 patients (21.87). SWL was successful in 14 patients (46.66%) in the first attempt and in 7 additional patients in the second intervention (23.33%).

**Conclusions:**

In this study, we showed different success rates for SWL and TUL because of the larger size of the stones. We achieved a success rate of 56.25% in the first attempt in the TUL group, and the overall success rate (after the second TUL) was 78.12%. In comparison, the SWL group had a success rate of 46.66% in the first attempt, and the overall success rate (after the second SWL) was 69.96%.

## 1. Background

A review of the related medical journals indicates that there is no definite evidence-based option for managing large proximal ureteral stones. The goal of treating ureteral calculi is to achieve complete stone clearance with minimal patient morbidity. Shock wave lithotripsy (SWL) and ureteroscopy have become standards of care for ureteral calculi. However, the optimal choice of treatment depends on various factors, including stone size, composition and location, clinical factors, equipment availability, and surgeon capability (1). Currently, many procedures such as transureteral lithotripsy (TUL), SWL, percutaneous nephrolithotripsy (PCNL), laparoscopic ureterolithotomy (LU), and open ureterolithotomy are used to treat this urological problem. Additionally, TUL with different sources of energy (i.e. laser, pneumatic, ultrasonic, and electrohydrolic) and with different modifications (i.e. urethral occlusion balloon catheter and stone cone) is widely used in the treatment of proximal ureteral stones (2-4) but its efficacy decreases in large stones. When stone removal is indicated, SWL and ureteroscopy (URS) are the two most commonly offered interventional procedures and they are both acceptable as first-line treatments. However, for stones < 10 mm, SWL at 90%, had a higher stone-free rate than URS (5). A percutaneous approach may also be indicated for large proximal ureteral stones, especially for large residual stones after PCNL and for impacted stones in hydronephrotic kidneys, as the percutaneous approach provides better results than URS for larger stones (6). LU has been suggested as a primary modality for large proximal ureteral stones and can be performed safely as a first-line procedure without increasing the complication rate compared with conventional URS. Although LU led to a prolonged operative time, a longer hospital stay, and greater blood loss, it has the advantage of a higher clearance rate in a single procedure (7). Ureterolithotomy, which has been abandoned in the era of advanced endourology and urolaparoscopy, is generally indicated for failed endourological procedures, particularly in centers that do not have a flexible ureteroscope or a laser lithotriptor, and in patients with larger stones (> 3 cm). Children are also candidates for open surgery, if specifically designed endourological equipment is not available (8). It is clear that although there are many effective treatment modalities for large proximal ureteral stones, there is no consensus about the modality of choice but the most common procedures are TUL and SWL.

## 2. Objectives

In this study, we tried to determine the most appropriate treatment plan for proximal ureteral stones larger than 12 mm by comparing two most commonly used procedures.

## 3. Patients and Methods

Between February 2005 and April 2011, 62 patients including 40 males and 22 females (mean age 39.5 years, range 19 to 64) with proximal ureteral stones larger than 12 mm (12-26 mm) and with a mean size of 17.64 mm were prospectively allocated randomly in 2 groups. Before the operation, ureterolithotomy was offered to the patients as the salvage procedure but never was needed in this study. The advantages, disadvantages, and possible side effects of each method were carefully explained to patients before obtaining informed consent. The success or failure of the procedure as well as the time needed for each procedure, cost, postoperative analgesic use, complications, stone clearance, and hospital stay were compared in the two groups.

In group A, 32 patients underwent URS with a semi-rigid wolf 8–9. 8F ureteroscope, and TUL was performed in successfully accessible cases. In nonaccessible cases, a 4.8F double-J stent was inserted blindly next to the stone, after unwanted pushed-back stones, or for large displaced fragments. Accessibility was defined as being able to reach the stone through the ureteroscope, and a successful outcome was defined as the patient being stone-free on radiography and ultrasound one month after the treatment. The procedure was performed under spinal anesthesia in group one. The sources of energy in the TUL group were ultrasonic and pneumatic.

In group two, 30 patients underwent SWL under intravenous sedation with pethidine as an outpatient procedure. The initial voltage of each shock wave was 13 kV, which was gradually increased to 18 kV. The maximum number of shock waves was limited to 4,500. In unsuccessful cases, repeat SWL or TUL was planned. Lack of success was defined as no change in the stone burden after the first postoperative X-ray and ultrasound one week after the operation, and a successful outcome was defined as a stone-free state one month after the procedure. Asymptomatic residual stones with a size of less than 5 mm were ignored. The lithotriptor used in the SWL group was the Dornier compact delta 2 lithotripter.

The indication for treatment was proximal ureteral stones larger than 12 mm (12–26 mm). Patients who could not tolerate the lithotomy position, younger than 18 years, had undergone coagulopathy, had concurrent renal and ureteral stones, were pregnant, or had sepsis were excluded from this study.

A preoperative conventional X-ray of the kidneys, ureter, and bladder (KUB) as well as ultrasound or excretory urography (IVP) were performed in all cases to determine the stone size and location and to estimate renal function and hydronephrosis. The postoperative image protocol included KUB and renal ultrasound to monitor the recovery of hydronephrosis and stone passage weekly. Treatment outcomes were determined based on evidence of being stone-free on KUB and ultrasound one month after the initial therapy.

The stone-free rates of the two methods were compared using Fisher’s exact test. Stone size, operation time, and age of the participants in the two groups were compared by an independent sample t-test. In addition, the cost in the two groups was analyzed in terms of the cumulative fees of preoperative evaluation, operation, perioperative monitoring, postoperative care, office visits, ancillary procedures, and re-treatment procedures.

## 4. Results

Stone access was successful in 28 patients and treatment was efficient in 18 patients (56.25%) in group one. For the patients with successful stone access but unsuccessful TUL, a DJ was inserted and a second ureteroscopic intervention was performed. The second intervention was successful in 7 additional patients (21.87). Double-J stents were also used in some cases with successful results to facilitate the recovery period. These stents were inserted in 20 patients because of the large stone burden, which enabled stone passage and prevented immediate postoperative complications, particularly in case of unsuccessful stone access or escaped stones in group one. The initial stone-free rate of the TUL group was 56.25% (18 of 32), and the final stone-free rate was 78.12% (56.25 + 21.87).

SWL was performed as a primary procedure in 30 patients. The initial stone-free rate for SWL was 46.66% (14 of 30), and in the second session it was successful in 7 more patients (23.33). Thus, the final stone-free rate in this group was 69.99%. The difference in the proportion of success between the two groups was significant (Table 1). There was no significant difference between the two groups’ mean scores with regard to operation time and stone size, although the difference between the two groups’ mean time of hospitalization was significant (Table 2).

**Table 1:** Comparison Two Group With Respect Final Success Frequency

	Final Outcome		Total	X Square	df	*P* value
	Failure, No.(%)	Success No.(%)				
TUL	7 (21.9)	25 (78.1)	32	1.644	1	0.2
SWL	11 (36.7)	19 (63.3)	30			
Sum	18 (29)	44 (71)	62			

**Table 2:** Comparison Two Groups With Respect Mean of Stone Size and Mean Time of Hospitalization

**Variables**	**Subjects, No.**	**Mean ± SD**	**t statistics**	**df**	**P value**
Stone size, mm			-0.109	60	0.913
TUL	32	17.59 ± 3.8264			
SWL	30	17.693 ± 3.3126			
Time of operation, h			0.053	59	0.958
TUL	31	48.55 ± 20,2			
SWL	30	48.33 ± 9.228			
Time of Hospitalization, h			11.379	60	0.0001
TUL	32	26.5 ± 9.228			
SWL	30	5.97 ± 3.643			

The cost of SWL in Iranian hospitals, particularly in nongovernmental hospitals, is definitely greater than that of TUL, even in the case of an ancillary procedure like DJ stent insertion. No significant complication was encountered in this study (i.e., hematuria, flank pain), except for low-grade fever of a limited duration (Chi-square = 3.847 df = 1 P-value = 0.05) and minor complications related to anesthesia (Chi-square = 9.526 df = 1 P-value = 0.02). The mean number postoperative office visits was higher in the SWL group (4 visits versus 2.2).

## 5. Discussion

The likelihood of the passage of a ureteral stone depends on stone size, location, configuration, and presence or absence of co-morbidities. Most stones ≤4 mm may pass spontaneously. For larger stones, there is a progressive decrease in spontaneous stone passage. The management of large proximal ureteral stones remains challenging for urologists (9). These stones are frequently associated with obstruction and deteriorated renal function. SWL as a modality of less invasive treatment has been advocated as the primary treatment modality, but its success rate is decreased for large proximal ureteral stones. In contrast, in patients with proximal ureteral stones larger than 10 mm, the treatment outcome after ESWL is not good if moderate to severe hydronephrosis is noted on ultrasonography. Alternative treatments, such as ureteroscopic lithotripsy, may be appropriate as an initial treatment or after the failure of one session of ESWL (10). Recent advances in the miniaturization of ureteroscopes, flexible ureteroscopes, and laser technology may encourage endoscopic interventions to solve this problem. Large migrated stones following PCNL may require percutaneous approach, and for many reasons, open ureterolithotomy and/or laparoscopy may be selected. SWL and URS have become standards of care for ureteral calculi. However, the optimal choice of treatment depends on various factors, including stone size, composition and location, clinical factors, equipment availability, and surgeon capability. SWL is the least invasive treatment for calculi of the upper urinary tract and is recommended as a first-line therapy. However, SWL has a variable success rate for large upper ureteral calculi (11).

Others have demonstrated that URS with laser lithotripsy achieves excellent results for upper ureteral calculi greater than 1 cm (2). Thus, this procedure should be considered as a first-line therapy for large proximal ureteral stones. Therefore, we see variable results and recommendations in different studies. Many investigators have compared these two modalities of treatment for large proximal ureteral stones. For example, Ziaee and coworkers have shown that SWL has sufficient capacity for the management of proximal ureteral stones 10 to 15 mm in size. Although URS tends to make patients stone-free faster because of the minimally invasive nature of SWL, patients still favored it over URS (12). Tawfick compared SWL and semi-rigid URS with lithoclasty and concluded that URS with lithoclasty can be considered as an acceptable treatment modality for large proximal ureteral calculi and can be considered as a first-line treatment of large proximal ureteral stones (13). The initial stone-free rate of the TUL group was 56.25% (18 of 32) and the final stone-free rate was 78.12% (56.25 + 21.87). The initial stone-free rate for SWL was 46.66% (14 of 30), and in the second session, it was successful in 7 additional patients (23.33), making the final stone-free rate 69.99%. The TUL group appeared to have better results, but the Chi-square test did not identify a significant difference between the expected and observed initial stone-free rates of the TUL and SWL groups (Chi-square = 0.153 , degrees of freedom = 1, and probability = 0.695). Cost analysis indicated that the cost of SWL in Iranian hospitals, particularly in nongovernmental hospitals, is definitely higher than that of TUL, even in the case of an ancillary procedure like DJ stent insertion. The results of the present study also indicate that except for cost, there was no significant difference between these two procedures in terms of other variables such as operation time, stone size, and side effects. Therefore, it could be strongly argued that SWL has the advantages of less invasiveness and shorter hospital stay, and TUL has the advantages of lower cost and faster stone-free state. Otherwise, there are no significant differences between these two procedures for other variables such as success rate, operation time, and side effects.
